# Developing, mature, and unique functions of the child’s brain in reading and mathematics

**DOI:** 10.1016/j.dcn.2019.100684

**Published:** 2019-07-26

**Authors:** Alyssa J. Kersey, Kathryn-Mary Wakim, Rosa Li, Jessica F. Cantlon

**Affiliations:** a Department of Brain and Cognitive Sciences, University of Rochester, Rochester, NY, USA; b Department of Psychology, University of Chicago, Chicago, IL, USA; c Neuroscience Graduate Program, University of Rochester Medical Center, Rochester, NY, USA; d Duke Institute for Brain Sciences, Duke University, Durham, NC, USA; e Department of Psychology, Carnegie Mellon University, Pittsburgh, PA, USA

**Keywords:** Mathematics, Reading, Natural viewing, fMRI, Early childhood

## Abstract

Cognitive development research shows that children use basic “child-unique” strategies for reading and mathematics. This suggests that children’s neural processes will differ qualitatively from those of adults during this developmental period. The goals of the current study were to 1) establish whether a within-subjects neural dissociation between reading and mathematics exists in early childhood as it does in adulthood, and 2) use a novel, developmental intersubject correlation method to test for “child-unique”, developing, and adult-like patterns of neural activation within those networks. Across multiple tasks, children’s reading and mathematics activity converged in prefrontal cortex, but dissociated in temporal and parietal cortices, showing similarities to the adult pattern of dissociation. “Child-unique” patterns of neural activity were observed in multiple regions, including the anterior temporal lobe and inferior frontal gyri, and showed “child-unique” profiles of functional connectivity to prefrontal cortex. This provides a new demonstration that “children are not just little adults” – the developing brain is not only quantitatively different from adults, it is also qualitatively different.

## 1 Introduction

During early childhood humans construct a variety of new concepts that lay the foundation for complex cognition (Carey, 1985). These first several years of life are also characterized by drastic changes in the structure of the brain (Giedd et al., 1999; Gogtay et al., 2004; Huttenlocher, 1990). A major focus of research over the last two decades has been to understand the relation between cognitive and neural development (Amso and Casey, 2006; Casey et al., 2005, 2000; Saxe et al., 2004; Shaw, 2007).

Patterns of functional brain development in children are characterized by their maturity. For instance, mature patterns are observed when children and adults rely on similar neural mechanisms or when they recruit the same regions of the brain for a particular task (e.g., Ansari and Dhital, 2006; Ansari et al., 2005; Cantlon et al., 2006; Golarai et al., 2007; Gomez et al., 2018; Hyde et al., 2010; Kersey and Cantlon, 2017a). In contrast, immature, developing patterns are identified within adult neural substrates when children and adults show differences in levels of amplitude, temporal pattern, location, or extent of activity (Ansari et al., 2005; Ansari and Dhital, 2006; Cantlon et al., 2011, 2006; Durston et al., 2006; Golarai et al., 2007). A third, underexplored pattern is “child-unique” neural activity in which children may show patterns of neural functioning that are not evident in adults.

Substantial research in cognitive development shows that during periods of early learning, children engage in a number of child-unique and often idiosyncratic strategies that might be supported by “child-unique” neural processes (Cornell, 1985; Ehri and McCormick, 1998; Fischer, 2011; Fischer and Koch, 2016; Mock et al., 2019; Portex et al., 2018; Siegler, 1996; Siegler and Jenkins, 1989; Vellutino, 1979). For example, as 6- to 8-year-old children are learning to add, they use a variety of inefficient strategies, including counting on all of their fingers and counting from one instead of from the larger addend (Siegler, 1996; Siegler and Jenkins, 1989). Similarly, children who are learning to read process letters, digits, and words independently of orientation, which results in mirror writing (Cornell, 1985; Fischer, 2011; Fischer and Koch, 2016; Portex et al., 2018), difficulties in distinguishing easily-confusable letters and numbers (Ehri and McCormick, 1998), and reading words backwards, such as reading “saw” as “was” (Vellutino, 1979). These spontaneous, child-unique strategies in reading and mathematics tend to emerge around 4 years of age and resolve by 8 years of age, coinciding with important periods of conceptual change in those domains.

Children’s unique behaviors suggest that the neural processes that underlie mathematics and reading acquisition in early childhood, particularly prior to important milestones of conceptual change, will differ from those of adults not only in degree but also in kind. The process of neural development could be one in which children gradually hone adult neural mechanisms for reading and mathematics by showing gradual change in the amplitude, temporal pattern, or extent of adult neural substrates (“developing” patterns of neural activity). More likely, however, the neural mechanisms of early mathematics and reading are also distinct in some way from those that underlie adult cognition and exhibit “child-unique” patterns of activity.

“Child-unique” neural activity likely takes many forms. For one, neural activity can be thought of as “child-unique” if the amplitude of activation during a controlled task is higher in children than in adults, that is if there is a *quantitative* difference in strength of neural activity in regions that are not recruited by adults (Ansari et al., 2005; Cantlon et al., 2011, 2009). Other forms of “child-unique” neural activity may represent *qualitative* differences in temporal patterns of neural activity during a particular task or during rest. For one, neural activity could be considered “child-unique” if children’s neural activity bears a temporal pattern that is more synchronous among children than among adults, which would suggest that children are using a particular brain region in ways that are similar to other children, but distinct from adults (Cantlon and Li, 2013). Finally, neural activity could qualify as “child-unique” if it bears a unique pattern of functional connectivity between brain regions in children compared to adults, such that children show evidence of connectivity among regions that do not function synchronously in adults (Dosenbach et al., 2010; Fair et al., 2009; Supekar et al., 2010, 2009). In these latter two cases, the regions that show “child-unique” patterns of neural activity may be used to the same degree in children and adults (i.e. children and adults may show similar amplitude of neural activity), but importantly differ qualitatively in the timecourse of neural activity.

An unexplored issue in cognitive neuroscience is to what extent the developing brain exhibits “child-unique” patterns of neural activity in reading and mathematics. Previous research suggests a functional dissociation between mathematics in parietal cortex and reading in temporal cortex, with overlapping recruitment of frontal cortex (Ansari et al., 2006; Bitan et al., 2007; Cantlon et al., 2009, 2006; Cantlon and Li, 2013; Emerson and Cantlon, 2012; Holloway and Ansari, 2010; Houdé et al., 2010; Lussier and Cantlon, 2017; Menon et al., 2000; Menon, 2014; Price et al., 2013; Price and Ansari, 2011; Rivera et al., 2005; Rosenberg-Lee et al., 2015, 2011; Turkeltaub et al., 2003; Yeatman et al., 2010). Recently, it was shown that this pattern of dissociation was evident within-subjects in children as young as seven (Evans et al., 2016). However, it is unclear whether this dissociation is present in younger children during important periods of conceptual change and to what extent “child-unique” temporal patterns of neural activity are evident in domain-specific regions that dissociate reading and mathematics versus domain-general regions, such as prefrontal cortex, that are recruited for both reading and math.

The objectives of the current study are two-fold: the first is to establish that the cortical networks related to reading and mathematics are functionally dissociable in early childhood, and the second is to test whether there are *qualitative* patterns of adult-like, developing, or “child-unique” neural activation within those cortical networks. Specifically, we test for systematic differences in temporal patterns between children and adults. Establishing the functional profiles and locations of “child-unique” vs developing vs adult-like activations in the brain is an important first step toward studying conceptual change in the human brain during childhood. Here we tested 4- to 8-year-old children and adults on a combination of controlled tasks and natural viewing tasks in the domains of reading and mathematics. Tasks were designed to ensure that the content of the tasks was understandable by all children and adults. Therefore, the mathematics tasks focused on counting and the meanings of symbolic numbers, and the reading tasks focused on word forms and grapheme-to-phoneme correspondences. We conducted traditional general linear model (GLM) analyses on neural amplitudes, and we conducted intersubject correlation and functional connectivity analyses on temporal patterns of neural activity. We tested our prediction that “child-unique” patterns of activation would distinguish the neural profiles of children from those of adults during reading and mathematics tasks. Then we tested whether “child-unique” activations emerge within domain-specific regions that show functional dissociations between reading and mathematics tasks, such as the temporal and parietal cortices, or in regions that have domain-general profiles, such as prefrontal cortex. Cognitive development research suggests that conceptual change in childhood is driven by interactions between general processes and core processes that test new rules on pre-existing concepts (Carey et al., 2015). Our hypothesis is that in childhood, reading and mathematics rely on interactions between children’s unique domain-general strategies (i.e., prefrontal uniqueness) and their core cognitive functions that are domain-specific in reading (i.e. temporal cortex) and mathematics (i.e., parietal cortex; Dehaene and Cohen, 2007).

## 2 Methods

### 2.1 Participants

42 children (4.11–8.77 years, mean age = 6.35 years, 17 girls) and 29 adults (18.44–28.09 years, mean age = 22.0 years, 16 women) were recruited to participate in this study. All participants had normal or corrected-to-normal vision and no history of neurological impairments. All participants or their parents provided written, informed consent in accordance with the University of Rochester Research Subjects Review Board.

### 2.2 Behavioral assessments

To identify relations between neural activity and cognitive development, children were administered the TEMA-3 (Ginsburg and Baroody, 2003) and TERA-3 (Reid et al., 2001), standardized Tests of Early Mathematics and Reading Abilities (one child who successfully completed the fMRI scan did not complete the TERA-3). The TEMA-3 provides one overall measure of math ability. The TERA-3 is composed of three subtests assessing children’s knowledge of the alphabet, reading conventions, and word meaning. We used the correlations between reading subtests scores and neural maturity to calculate an overall measure of the relation between reading ability and neural maturity (see Methods 2.5 for more details). Due to concerns about ceiling effects, children who scored above the 99^th^ percentile for their age were excluded from the corresponding correlation analysis (TEMA-3: n = 3, TERA-3: n = 3, 0, & 3 for subtests 1, 2, & 3). Raw scores were used for all analyses.

### 2.3 fMRI session

Prior to scanning, children completed a 30-minute mock scanner session during which they practiced staying still and practiced the tasks. During scanning, children’s heads were stabilized with headphones, foam padding, and medical tape. Adults received verbal instructions to remain motionless and were reminded of task instructions prior to each run.

#### 2.3.1 MR parameters

Neural activity was measured via whole-brain BOLD imaging on a 3-Tesla Siemens MAGNETOM Trio scanner with a 12-channel head coil at the Rochester Center for Brain Imaging. High-resolution structural T1 contrast images were acquired using a magnetization prepared rapid gradient echo pulse sequence at the start of each session [repetition time (TR) =2530 ms, echo time (TE) =3.44 ms, flip angle = 7, field of view (FOV) = 256 mm, matrix = 256 × 256, 192 or 176 [depending on head size] 1 × 1 × 1 mm sagittal left-to-right slices].

An echo-planar imaging (EPI) pulse sequence was used for T2* contrast (TR =2000 ms, TE =30 ms, flip angle = 90 degrees, FOV = 256 mm, matrix 64 × 64, 30 axial oblique slices, parallel to the AC-PC plane, voxel size = 4 × 4 × 4 mm). For the children, we also collected a second series of data with retrospective motion correction applied to the original EPI series using built-in Siemens software for online motion correction. Functional scans consisted of 3 paradigms and a no-stimulation resting state scan (described below). Total scanning time was approximately 1 h. Children were excluded from the analyses based on excessive head motion as described below in sections 2.3.2 – 2.3.5 (> 3 mm volume-to-volume motion and/or > 5 mm displacement from the start of the session, based on raw EPI data that had not been corrected for motion).

#### 2.3.2 Natural viewing paradigm

Children and adults watched an 11-minute 35-second montage of educational movie clips. Individual clips ranged from 12.5 to 32.4 s and were edited to form one continuous video. Topics included counting, word reading, and phonics. Participants were instructed to remain motionless and observe the movie. No instructions were given to fixate or restrict eye movement.

All children and adults completed this scan. Children were excluded due to excessive head motion (> 3 mm volume-to-volume notion and/or > 5 mm displacement from the start of the session; n = 7 children). 6 adults who watched an alternate version of the movie were also excluded. Data from 35 children and 23 adults were included in the final sample.

#### 2.3.3 Word localizer controlled task

Neural responses to picture of words, scrambled words, and shape strings were measured during a 1-back task. White stimuli (words, scrambled words, or shapes) were presented against a black background for 500 ms. Participants were instructed to press a button when presented with the same picture twice in a row. Stimuli were presented in 46 miniblocks per run and each miniblock consisted of 1–12 trials. Participants were instructed to fixate on an image of a snowflake for 2 s between consecutive same-category miniblocks and for 12 s between consecutive different-category miniblocks. Participants viewed 260 stimuli in each of the two 4.55-minute scans.

37 children (4.11–8.77 years, mean age = 6.49 years, 14 girls) and all 29 adults completed 1 (n = 2 children) or 2 (n = 35 children, 29 adults) runs of this task. Data were excluded from children due to excessive head motion (> 3 mm volume-to-volume motion and/or > 5 mm overall motion; n = 17 runs from 14 children), experimental error (n = 2 runs from 1 child), or failure to provide any responses during the task (n = 6 runs from 3 children). Data from 28 children and 29 adults were analyzed after exclusions.

#### 2.3.4 Number localizer controlled task

Participants’ neural responses to pictures of numbers, faces, tools, and words were measured during a matching task. Stimuli were presented two at a time as gray-scale (faces) or white (letters, tools, Arabic numerals/dot arrays) images against a green background, one stimulus on either side of the screen (Kersey et al., 2016). Participants were instructed to compare within-category stimuli and to press a button when the stimuli matched. Faces and tools were compared across orientation: frontal shot vs oblique (faces) or upright vs rotated views (tools). Number and word stimuli were compared across notation: Arabic numerals vs dot arrays (numbers), or normal vs mirrored views (words). Participants completed 36 trials during the 4-minute run.

32 children (4.11–8.77 years, mean age = 6.60 years, 11 girls) and all 29 adults completed 2 runs of the number localizer task. Data were excluded from children due to excessive head motion (> 3 mm volume-to-volume motion and/or > 5 mm overall motion; n = 21 runs from 15 children). The final sample consisted of data from 26 children and 29 adults.

#### 2.3.5 Resting state scan

A subset of participants (34 children, 4.11–8.77 years, mean age = 6.41, 11 girls; and all 29 adults) completed a 5-minute resting state scan. Resting state neural activity was measured as children and adults lay passively with their eyes closed. Eleven children were excluded due to excessive motion (> 5 mm overall motion), leaving a final sample size of 23 children and 29 adults.

### 2.4 Preprocessing of fMRI data and thresholding

fMRI data were processed in BrainVoyager 2.8.1.4 (Goebel et al., 2006) using in-house scripts. Functional data were registered to high-resolution anatomy images for each participant in native space. Subsequent processing of functional data included slice scan time correction (cubic spline interpolation), linear-trend removal in the temporal domain (cutoff: 2 cycles within the run), motion correction with respect to the first volume in the first run of each task, and spatial smoothing (a Gaussian spatial filter with a 6 mm full-width half-maximum was applied to each volume). Functional and anatomical volumes were then transformed to Talairach space using piecewise affine transformation after manually aligning stereotactic axes to anatomical loci. Analyses were performed on processed data in Talairach space.

Primary analyses focused on functional data from the natural viewing paradigm. Although children’s movement for this task was fairly minimal, it was slightly higher than adults’ motion (children included in analyses: translation = 0.79 mm average, sd = 0.55 mm, rotation = 1.15 degrees average, sd = 0.81 degrees; adults: translation = 0.53 mm average, sd = 0.37 mm translation, rotation = 0.68 degrees average, sd = 0.46 degrees; calculated on raw EPI series following Grill-Spector et al., 2008). Because removing higher-motion data through the process of scrubbing reduces the overall power of the primary intersubject correlation analysis and leads to varying degrees of freedom across comparisons, we accounted for the difference in motion in two other ways without removing any frames of data. First, we used the online motion corrected BOLD data for the children, and then we regressed frame-wise displacement (FD) across their brains to control for sudden changes in volume-to-volume head motion. FD was calculated by summing the absolute values of the derivatives of six motion predictors. Rotational displacements were then translated to millimeters and projected onto a sphere with a 50 mm radius (Power et al., 2012). These values were then regressed timepoint by timepoint to reduce any effects of sudden changes in intensity due to motion.

The threshold for the following analyses was voxel-wise p < 0.01, cluster corrected to p < 0.05. For some analyses, stricter thresholds were used to better separate the regions that showed the strongest differences between groups and the most similarity within each group. All between-group comparisons of adults vs children were conducted at voxel-wise p < 0.005, and all analyses of within-group neural similarity were conducted at a strict threshold of t > 5.69 (determined based on a threshold of p < 0.00001 for the group of 23 adults).

### 2.5 Intersubject correlation analyses (natural viewing & resting state data)

Natural viewing and resting state data were analyzed using an intersubject correlation approach to obtain measures of “neural similarity” (Cantlon and Li, 2013; Hasson et al., 2004). Intersubject correlations for the natural viewing data were performed by using the timecourse (the entire 11-minute video) of each voxel for each participant as a predictor for activation of the corresponding voxel in every other participant’s brain. Intersubject correlations for the resting state data were calculated ignoring the first 6 volumes to allow the scanner signal to stabilize in the absence of any task-driven neural activity. Three sets of correlations were calculated: 1) adults compared to every other adult, 2) children compared to every other child, and 3) children compared to every adult. The within-group comparisons of adults to other adults and children to other children are referred to as maps of “neural similarity.” The comparison of children to adults is a special type of neural similarity because it represents how “adult-like” each child’s functional timecourse appears. Therefore, we instead refer to these maps as maps of “neural maturity.” First, each participant’s functional data were correlated with that of every other participant to produce paired r-maps representing the timecourse similarity to each participant at each voxel. Then, within the three sets of intersubject correlations, each individual’s paired r-maps were averaged to obtain one measure of similarity that represents the mean similarity of that participant to the rest of the comparison group. This resulted in maps that represent the average similarity of 1) each adult to the group of adults, 2) each child to the group of children, and 3) each child to the group of adults. These maps were then transformed to t-maps using Fisher transformations.

Next, we identified profiles of “child-unique”, developing, and adult-like neural activity during natural viewing by conducting between group t-tests on the neural similarity maps within regions that showed significant group-level similarity (t > 5.69) and did not show group differences in Signal-to-Noise ratio (SNR). Signal-to-Noise ratio for the natural viewing data was calculated by dividing the mean signal intensity of each voxel by its standard deviation (Murphy et al., 2007; Simmons et al., 2010). Between group *t*-tests across neural similarity maps and SNR were conducted at the same threshold (p < 0.005). Regions were identified as “child-unique” if the neural similarity between children was greater than the neural similarity between adults (t(56) > 2.92, p < 0.005, corrected), and if there was significant similarity among children (*t*-test of child-to-child similarity maps vs 0: t(34) > 5.69, p < 0.000002). Regions that showed higher SNR in children were excluded from the identification of “child-unique” regions (excluded t(56) > 2.92, p < 0.005). Developing regions were identified as regions that showed greater similarity among adults than among children compared to adults, showed significant similarity among adults, and did not show greater SNR for adults compared to children (*t*-test of adult-to-adult vs child-to-adult similarity maps: t(56) > 2.92, p < 0.005, corrected; adult-to-adult maps vs 0: t(22) > 5.69, p < 0.000010; SNR: excluded t(56) > 2.92, p < 0.005). Adult-like regions of the brain were regions that did not show a significant difference between child-to-adult and adult-to-adult similarity, showed significant similarity at both the adult-to-adult and child-to-adult levels, and did not show differences between child and adult SNR (adult-to-adult maps == child-to-adult maps: t(56) < 2.92, p > 0.005, corrected; adult-to-adult maps vs 0: t(22) > 5.69, p < 0.000010; child-to-adult maps vs 0: t(34) > 5.69, p < 0.000002; SNR: excluded t(56) > 2.92, p < 0.005). To confirm that the patterns of “child-unique” and developing neural activity were not artifacts of motion, we conducted a regression analysis in each region that tested for a difference in neural similarity over and above effects of motion. These analyses revealed that motion could not explain the differences in similarity between child and adults (Supplement 1).

These same steps were also applied to the resting state data. Intersubject correlations and SNR maps were computed for the resting data. The thresholds applied to these analyses were consistent with those applied to the primary analyses of the natural viewing data (“Child-Unique”: *t*-test of child-to-child > adult-to-adult similarity maps: t(50) > 2.94, p < 0.005; *t*-test of child-to-child similarity maps vs 0: t(22) > 5.69, p < 0.000010; excluding regions from *t*-test of child > adult SNR: t(50) > 2.94, p < 0.005; Adult-like: adult-to-adult maps == child-to-adult maps: t(50) < 2.94, p > 0.005; adult-to-adult maps vs 0: t(28) > 5.69, p < 0.000004; child-to-adult maps vs 0: t(22) > 5.69, p < 0.000010; excluding regions from *t*-test of adult vs child SNR: t(56) > 2.92, p < 0.005; Developing: *t*-test of adult-to-adult > child-to-adult similarity maps: t(50) > 2.94, p < 0.005; adult-to-adult maps vs 0: t(28) > 5.69, p < 0.000004; excluding regions from *t*-test of adult > child SNR: t(50) > 2.94, p < 0.005).

Finally, following (Cantlon and Li, 2013), we tested for correlations between neural maturity of natural viewing and cognitive measures across the brain. Correlation analyses were conducted between each measure of cognition and neural maturity in every voxel of the brain. We collapsed across the correlations with the reading subtests (3 measures) to provide a comprehensive view of the relation between neural maturity and reading versus mathematics (1 measure).

### 2.6 GLM analyses (natural viewing & controlled localizer data)

Data from the natural viewing and localizer runs were analyzed using three general linear models (GLMs): one for the natural viewing movie, one for the number localizer, and one for the word localizer. Data from adults and children were combined into a single GLM, and experimental events were convolved with a standard dual hemodynamic response function. The GLM for the natural viewing task included 2 predictors of interest corresponding to math clips and reading clips. The GLM for the word localizer included 3 predictors of interest corresponding to the word trials, scrambled word trials, and shape trials. The GLM for the number localizer included 4 predictors corresponding to the 4 conditions of interest (number trials, face trials, tool trials, and word trials). All GLMs also included 6 predictors of no interest that corresponded to the motion parameters obtained during pre-processing. The GLMs for the controlled, localizer tasks contained an additional predictor of no interest that corresponded to button presses. Random-effects analyses were used to analyze the data.

### 2.7 Functional connectivity analyses (natural viewing data)

**Fig. 1:**
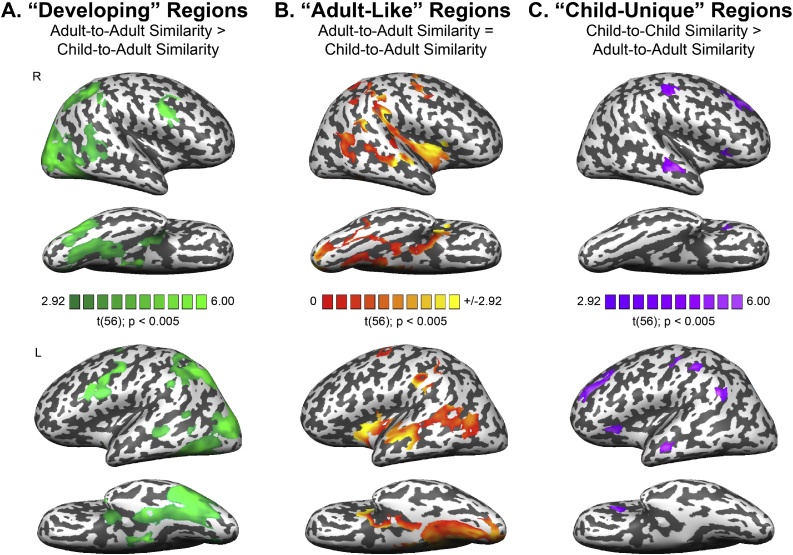
**Functional similarity between adults and children during natural viewing.** Regions identified as having Developing (A), Adult-like (B), or “Child-Unique” (C) patterns of neural activity based on the results of the between-group t-tests of neural similarity. Regions that showed developmental differences in signal-to-noise ratio (t(56) > 2.92, p < 0.005) or did not show significant within-group neural similarity (t(22) or t(34) < 5.69) were masked from the analysis (see methods, 2.5 Intersubject Correlations for more detail and see Supplement 2 for a table of Developing and “Child-Unique” regions). Results are displayed with an arbitrary cluster threshold of 50 mm^2^.

Functional connectivity analyses were conducted on the natural viewing data using the nine “child-unique” regions displayed on the surface rendering in Fig. 1C as seed regions. Time series from all voxels within each seed were averaged to create a single time series for each region. This time series was then correlated with every other voxel in each participant’s functional dataset. We used linear regression to reduce the influence of nuisance factors that were unrelated to neural activity. Specifically, we regressed the global mean timecourse and 6 motion parameters for all participants (following Emerson and Cantlon, 2012). For each of the nine sets of functional connectivity (one per seed region), we tested for regions that showed 1) strong connectivity in children (average r across children for 348 timepoints: r(346) > 0.25), and 2) stronger connectivity in children than adults as revealed by whole-brain regressions that test for effects of age-group over and above effects of motion (age-group predictor: t(54) > 2.93, p < 0.005). Regressions were conducted in MATLAB using the “fitlm” function with functional connectivity as the dependent variable and with age group (children vs adults), translation, and rotation as the independent predictor variables.

## 3 Results and discussion

### 3.1 Patterns of neural similarity between children and adults

We first measured voxel-by-voxel intersubject similarity from 23 adults and 35 children in the critical ages of 4 to 8 years during natural viewing of educational television clips about math and reading (Cantlon and Li, 2013; Hasson et al., 2004). To test for evidence of profiles of developing, adult-like, and “child-unique” neural activity, we conducted between group t-tests on the similarity maps within regions that showed significant group-level similarity (see Methods 2.5 “Intersubject Correlation Analyses”). Developing regions were identified by testing for greater adult-to-adult similarity than child-to-adult similarity. These patterns were found bilaterally in intraparietal sulcus, inferior frontal gyrus, middle and superior temporal gyri, inferior temporal cortex, and occipital cortex (Fig. 1A, Supplement 2). Adult-like regions did not show a significant difference between child-to-adult and adult-to-adult similarity but showed significant similarity at both the adult-to-adult and child-to-adult levels. These patterns were identified in bilateral superior temporal gyrus and in medial regions throughout the brain (Fig. 1B). Finally, “child-unique” patterns of activity were defined as regions that showed greater similarity among children than among adults and included left angular gyrus, bilateral postcentral gyrus, bilateral inferior frontal gyrus and insula, superior and middle frontal gyri, and anterior temporal lobe (Fig. 1C, Supplement 2). Regions that showed “child-unique” and developing patterns of neural activity are reported in a table in Supplement 2.

Importantly, a comparison of these results to a “no stimulation” resting state condition revealed that these patterns were not present at rest. Following the same approach as taken with the natural viewing data, we tested for patterns of “child-unique”, developing, and adult-like neural activity during rest by conducting between-group t-tests on resting state neural similarity and removing regions that showed differences in SNR or did not show group-level synchrony. In contrast to the results from the natural viewing task, there were no regions that were identified as “child-unique”, developing, or adult-like during rest (no regions of at least 10 contiguous voxels). This confirms that the patterns of neural activity identified by the natural viewing data are driven by functional processing of the task content. Because these same patterns did not emerge for resting state data, the patterns of “child-unique” neural activity cannot be due to general differences between children and adults. Therefore, the regions identified as developing, adult-like, and “child-unique” (Fig. 1, Supplement 2) represent true patterns of neural activity and are not artifacts of baseline (resting state) neural activity or differences in signal-to-noise ratio. Regression analyses revealed that these patterns are not the result of differences in motion (Supplement 1).

### 3.2 Cortical networks for reading and mathematics

**Fig. 2:**
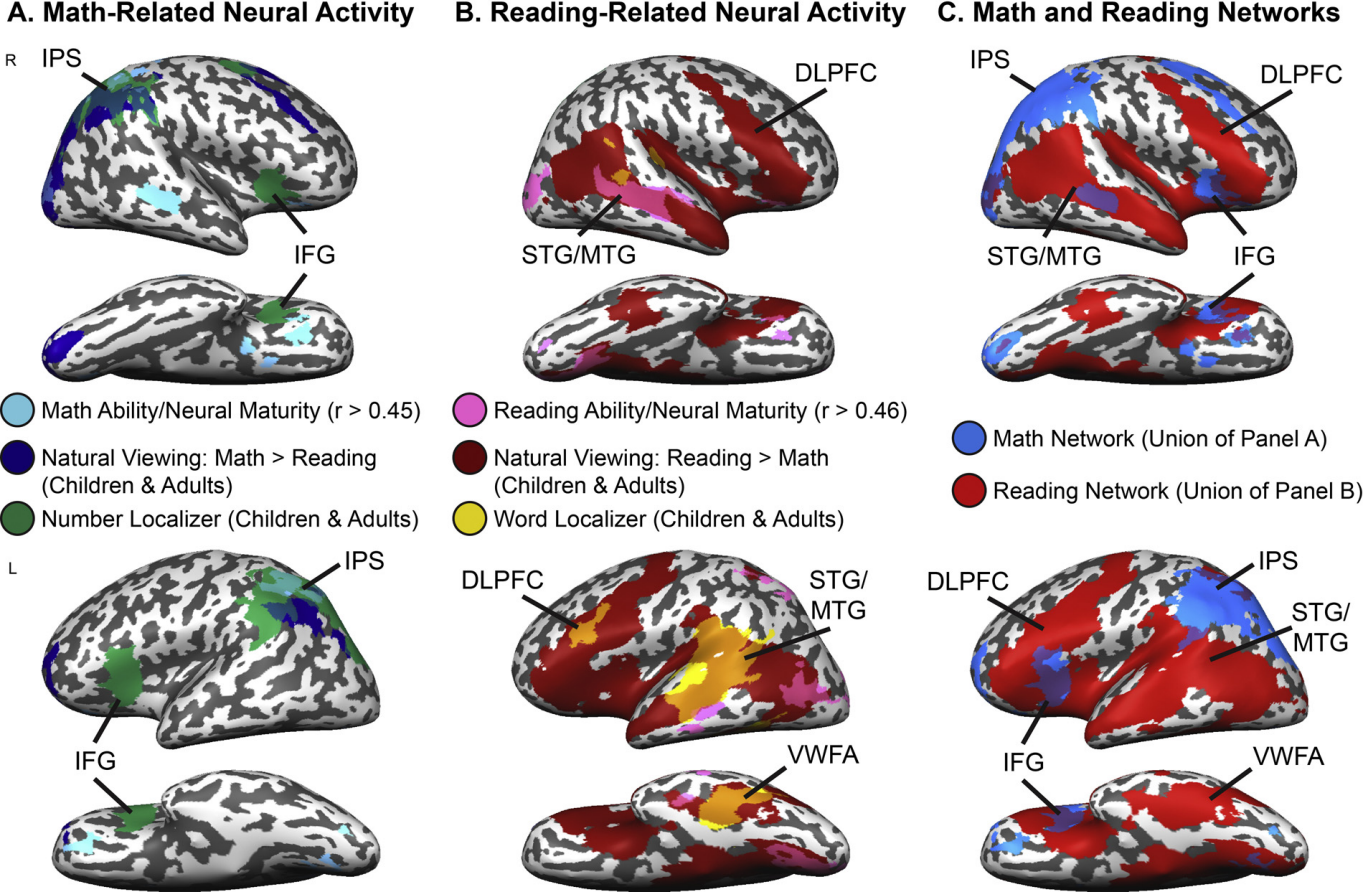
**Functional networks for mathematics and reading.** Analyses that identify math-related neural activity (A) and reading-related neural activity (B). Analyses in panels A and B draw on data from the natural viewing tasks (correlations between neural maturity and cognitive skills in light blue and pink, GLM analysis of mathematics video clips vs reading video clips in navy and red) and on data from the controlled localizer tasks (number task in green, word task in yellow). Supplement 3 presents a complete list of regions in these panels. Panel C shows the union of the analyses from Panel A in blue (the “mathematics network”) and the union of analyses from Panel B in red (the “reading network”). All results were significant at a voxel-wise p < 0.01, cluster corrected to p < 0.05. Maps are displayed with an arbitrary cluster threshold of 25 mm^2^. IPS = intraparietal sulcus, IFG = inferior frontal gyrus, STG/MTG = superior/middle temporal gyrus, DLPFC = dorsolateral prefrontal cortex, VWFA = visual word form area (For interpretation of the references to colour in this figure legend, the reader is referred to the web version of this article.).

Next, we identified cortical networks for reading and mathematics as the union of three sets of analyses that isolated reading-related and mathematics-related neural activity (see Supplement 3 for a table of all regions). First, we identified regions that showed a relation between patterns of neural activity during natural viewing and children’s cognitive development. Following Cantlon and Li (2013), we tested for correlations between neural maturity (child-to-adult similarity) and math and reading abilities. Relations between neural maturity and math scores were identified in bilateral parietal and occipital cortices (Fig. 2A, light blue; r(30) > 0.45, p < 0.01, corrected). In contrast, correlations between neural maturity and reading were observed in bilateral middle temporal gyrus, occipital cortex, and the left ventral temporal cortex (Fig. 2B, pink; TERA 1: r(29) > 0.46; TERA 2: r(32) > 0.44; TERA 3: r(29) > 0.46; all p < 0.01, corrected, the pink represents the union of the results for the three subtests). One region of the right middle temporal gyrus (MTG) showed correlations with both reading and math ability, indicating that neural maturity of the right MTG relates to the development of both reading and mathematics.

Then, we identified regions that were involved in processing each type of clip (reading vs math) by conducting a traditional random effects GLM analysis on the natural viewing data from children and adults. A contrast of reading clips vs math clips revealed that large regions of frontal and temporal cortices were involved in processing the reading clips (Fig. 2B, red), whereas regions of parietal cortex and angular gyrus were recruited during the math clips (Fig. 2A, navy; t(57) > 2.66, p < 0.01, corrected).

Finally, two traditional functional localizer fMRI tasks were used to identify regions that showed preferences for the foundational systems associated with math and reading: numbers and words. Number-preferring regions were identified as those that showed greater activation for matching cross-notation number stimuli (an Arabic numeral and an array of dots) than for matching face, word, or tool stimuli (Kersey et al., 2016). Children and adults showed number-preferring activation in bilateral intraparietal sulcus and bilateral inferior frontal gyrus (Fig. 2A, green; t(54) > 3.48,p < 0.01, corrected). Word-preferring regions were identified as those that showed greater activation to word stimuli than scrambled word stimuli during a 1-back task and were evident bilaterally in the superior temporal cortex and in left ventral temporal cortices (Fig. 2B, yellow; t(56) > 3.47, p < 0.01, corrected).

**Fig. 3:**
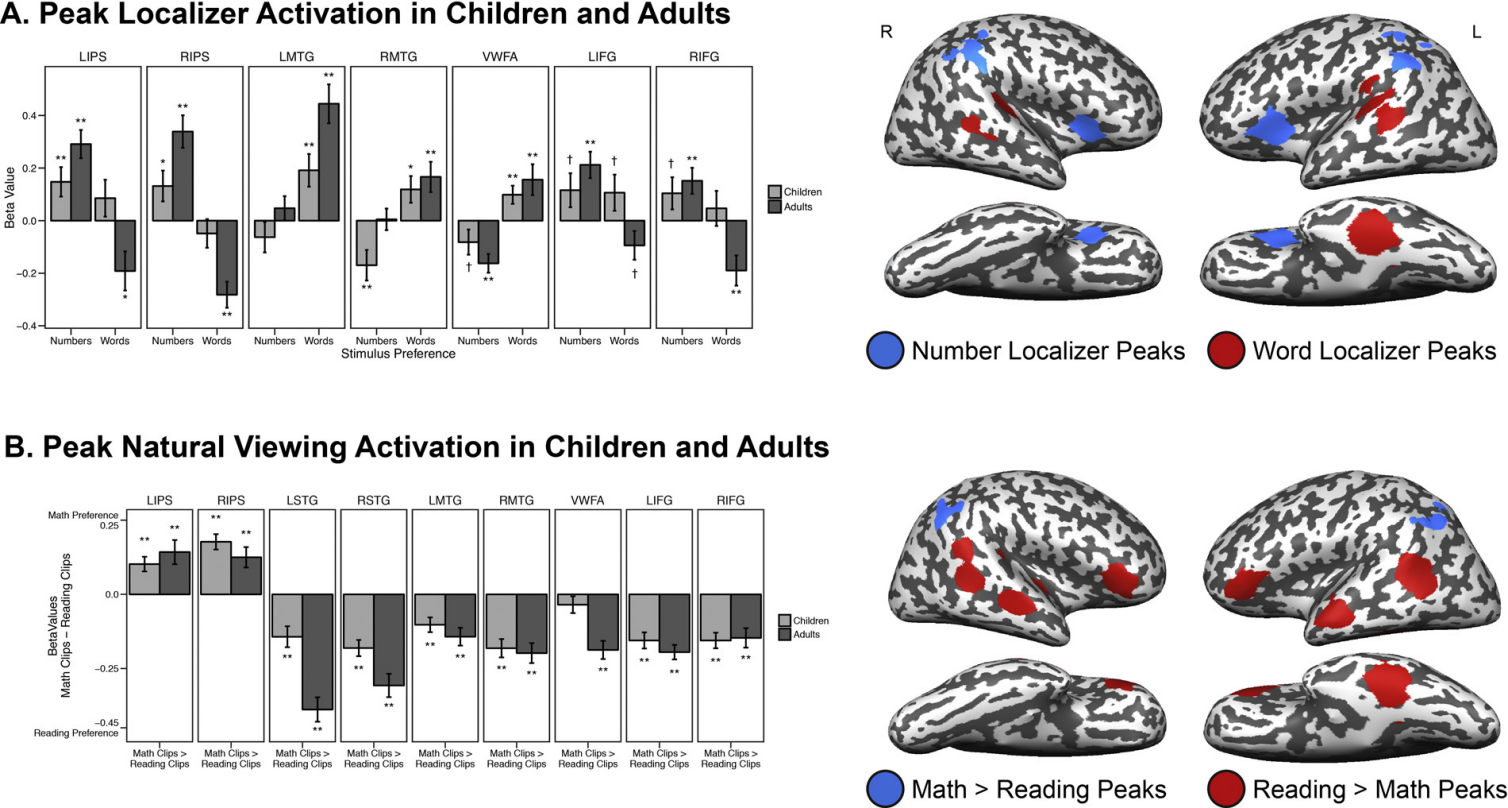
**Region of Interest analyses for the localizer (A) and the natural viewing (B) GLMs.** Sphere ROIs were created using the peaks from the GLM analyses in left (L) and right (R) intraparietal sulcus (IPS), middle and superior temporal gyri (MTG/STG), fusiform gyrus (VWFA, visual word form area), and inferior frontal gyrus (IFG). T-tests of beta values versus 0 for adults and for children show that the dissociation between mathematics in parietal cortex and reading in temporal cortex is evident in children alone. ** p < 0.01, * p < 0.05, † p < 0.10 Sphere ROIs were projected from volume space to surface space for consistency with other figures.

Across these three independent analyses on data from both the natural viewing and controlled tasks, we found a dissociation between math and reading in parietal vs temporal regions of cortex (Fig. 2C). Consistent with previous work, we saw that parietal cortex, namely the IPS and the angular gyrus, and the inferior frontal gyrus were involved in processing math content (Ansari et al., 2006; Cantlon et al., 2009, 2006; Cantlon and Li, 2013; Emerson and Cantlon, 2012; Holloway and Ansari, 2010; Lussier and Cantlon, 2017; Menon, 2014; Menon et al., 2000; Price et al., 2013; Price and Ansari, 2011; Rivera et al., 2005; Rosenberg-Lee et al., 2015, 2011). In contrast, reading primarily recruited regions in temporal and frontal cortices (Bitan et al., 2007; Evans et al., 2016; Turkeltaub et al., 2003; Yeatman et al., 2010). A post-hoc analysis of beta values from peaks in parietal and temporal cortices confirmed that this dissociation was evident even when looking at data from just the children (Fig. 3 and Supplement 3). This shows that the domains of reading and math are functionally dissociable in an adult-like manner as early as 4 years of age.

One exception to this dissociation was the overlap between math and reading in the right middle temporal gyrus (Fig. 2A). Although the right middle temporal cortex (MTG) has been implicated in phonological processing (Boets et al., 2013) and linked to reading ability (Conant et al., 2014), this is not a region that is typically recruited for math and numerical processing (Arsalidou et al., 2017; Kersey and Cantlon, 2017b; Peters and Smedt, 2018). In fact, a direct contrast of neural activation related to reading vs math during natural viewing indicated that this region was more strongly recruited for reading. However, our results also demonstrate that the individual differences in neural maturity of right MTG that are related to math acquisition are also related to reading acquisition, which suggests that maturation of this region may relate to broader conceptual change. Thus, while activation in MTG might be higher for reading than math, neural maturity of MTG activation is related to both reading and math.

#### 3.2.1 Conjunction of patterns of neural similarity and functional networks

**Fig. 4:**
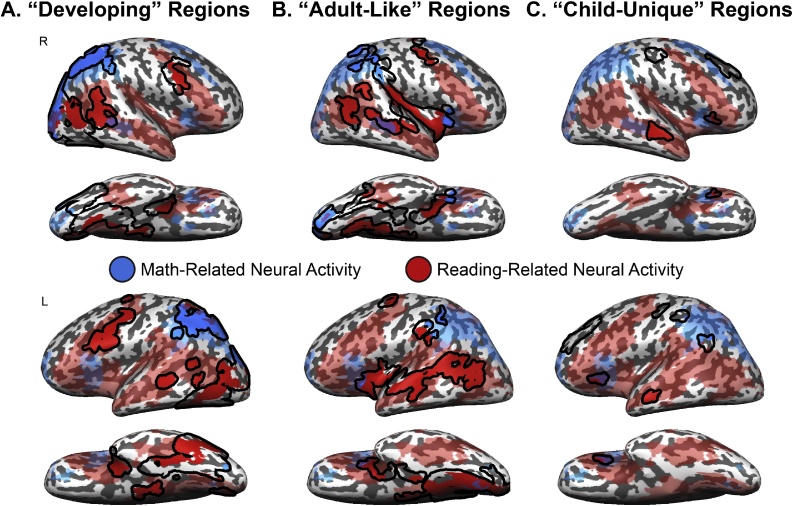
**Conjunction of the functional networks for reading vs math from**Fig. 2**C and the 3 patterns of neural similarity from**Fig. 1. All regions classified as Developing, Adult-like, or “Child-Unique” are outlined. The conjunction is shown by the colors within the outlined regions. The full mathematics (blue) and reading (red) networks from Fig. 2C are overlaid for visualization purposes (For interpretation of the references to colour in this figure legend, the reader is referred to the web version of this article.).

Next, we investigated the functions of the developing, adult-like, and “child-unique” patterns of neural activity by testing for conjunctions between those profiles of neural similarity (Fig. 1) and the math and reading networks (Fig. 2C). “Child-unique” patterns of neural activity converged with the reading network in bilateral anterior temporal lobe and with both the math and reading networks in bilateral inferior frontal gyrus (IFG; Fig. 4C). Previous work has found greater recruitment of the IFG for number and mathematical processing in children compared to adults (Ansari et al., 2005; Ansari and Dhital, 2006; Battista et al., 2018; Cantlon et al., 2009; Holloway and Ansari, 2010; Kersey and Cantlon, 2017b; Lussier and Cantlon, 2017; Rivera et al., 2005). The current finding suggests that this region not only shows differences between children and adults in the strength of math-related neural activity during controlled tasks as reported by previous research, but it also shows different temporal patterns during naturalistic processing of mathematical content. The reading and math networks also converged with profiles of developing neural activity (Fig. 4A) and adult-like neural activity (Fig. 4B). Taken together this indicates that within the math and reading networks, there are patterns of neural activity that fit all three profiles of neural simliarity.

Several of the regions that displayed “child-unique” patterns of neural activity did not show differential neural activity in favor of either reading or math. One possibility is that these regions are integral to many domains and show consistent levels of neural activity across many stimulus types. In this case, we may see evidence of communication with regions of the math and reading networks. Another possibility is that these regions of the developing brain show strong functional connectivity with each other at a level that is over and above what is observed in the adult brain.

### 3.3 Functional connectivity analyses

To better understand how “child-unique” regions function in the developing brain, we calculated whole-brain functional connectivity during natural viewing using each of the nine “child-unique” regions in Fig. 1C as seeds. Broadly, we found that “child-unique” regions showed strong functional connectivity to most of the brain, including regions within the mathematics and reading networks (average r-value across children: r(346) > 0.25, p < 0.0001). “Child-unique” regions showed particularly strong connectivity with their opposite hemisphere counterparts (functional homotopy), suggesting possible cross-talk between corresponding “child-unique” regions. Strong functional homotopy in childhood has been observed in previous studies of resting state functional connectivity (Anderson et al., 2011; Zuo et al., 2010) and is consistent with task-based studies that find stronger bilateral recruitment of regions in childhood, which is often thought to reflect immaturity of networks that will become lateralized with age or experience (Bunge et al., 2002; Smyser et al., 2010; Wood et al., 2004). One intriguing possibility is that functional homotopy is an important characteristic of regions that show consistency across children. In other words, these regions not only have similar timecourses across children, but also more similar timecourses to their contralateral counterpart in early childhood.

**Fig. 5:**
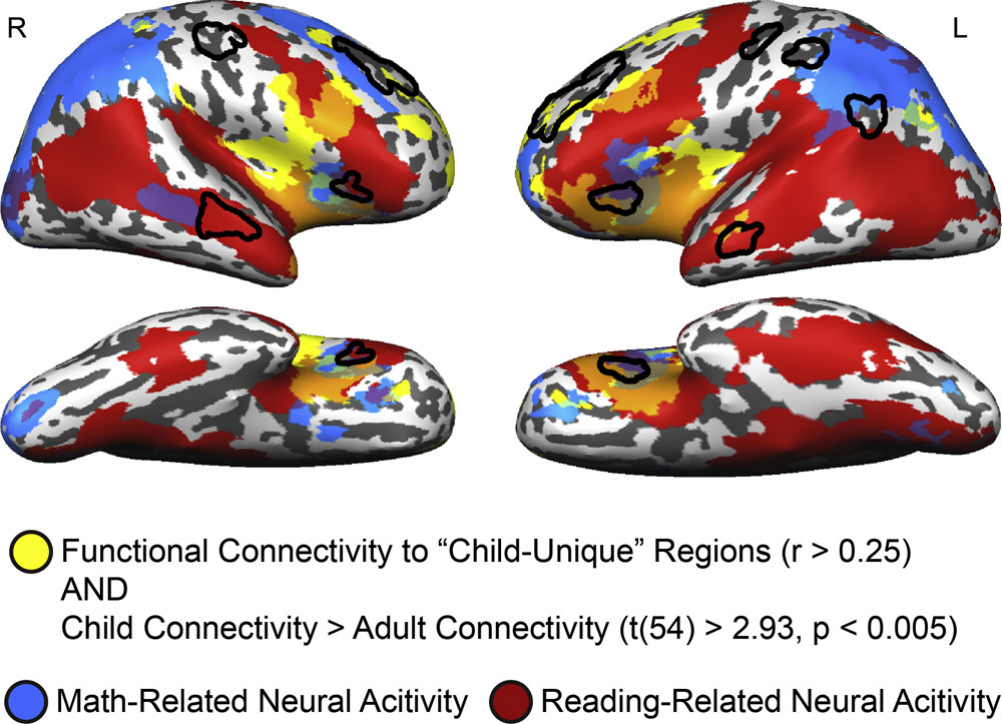
**“Child-unique” functional connectivity.** Reading network (red), math network (blue), and the conjunction of regions where 1) children showed strong functional connectivity to any “child-unique” seed region and 2) functional connectivity in children was greater than functional connectivity in adults (yellow). Outlined regions show the seed regions (the “child-unique” regions from Fig. 1C). Notes: the conjunction maps were calculated separately for each seed and results are displayed with an arbitrary cluster-threshold of 50 mm^2^ (For interpretation of the references to colour in this figure legend, the reader is referred to the web version of this article.).

To determine whether these functional connectivity patterns were “child-unique”, we compared maps of children’s and adults’ functional connectivity to “child-unique” regions by using linear regression to control for motion (see Methods for details). Within regions that showed strong functional connectivity in children (r > 0.25), children showed stronger connectivity than adults throughout the prefrontal cortex (t(54) > 2.93, p < 0.005; Fig. 5, yellow, see Supplement 4 for a table of results). This effect was primarily driven by functional connectivity with the left and right inferior frontal gyri, which showed stronger functional homotopy and stronger connectivity to nearby regions within the ipisilateral hemisphere. As described in Methods, we controlled for differences between children and adults in head motion. We also compared the regression results to results obtained using the conventional approach of censoring or "scrubbing" the data (Power et al., 2012; Satterthwaite et al., 2019, 2012; van Dijk et al., 2012). Results were similar across the two analyses (Supplement 5). This indicates that differences in head motion are unlikely to be the cause. Instead, this finding is theoretically interesting for three reasons.

First, the left and the right inferior frontal gyri were the only two regions that displayed patterns of “child-unique” neural activity that overlapped with both the reading and math networks, indicating that they support cognitive processing in both domains. Second, prefrontal cortex is broadly implicated in executive functions such working memory and cognitive control (Bunge and Zelazo, 2006; Casey et al., 1997, 1995; Cohen et al., 1994; Perone et al., 2018; Shallice and Burgess, 1991; Zelazo, 2015), which are thought to be important for transitioning to more complex conceptual representations (Carey et al., 2015). Finally, structural and functional maturity of prefrontal cortex are considerably protracted compared with sensory cortices and posterior functional networks (Bunge et al., 2002; Casey et al., 2005, 2000; Gogtay et al., 2004; Huttenlocher, 1990). Taken together, one potential implication is that the inferior frontal gyrus acts as a hub between domain-general executive processes and core knowledge systems. This interaction between domain-general executive processes and core knowledge systems has been described as the catylst for conceptual change in early childhood (Carey et al., 2015). If this is the case, then the “child-unique” patterns of neural activity that were evident during natural viewing might reflect child-unique strategies that are important precursors to conceptual development in mathematics and reading.

## 4 Summary and conclusions

Here, we have identified developing, adult-like, and “child-unique” patterns of neural activity during a naturalistic task. We also identified an adult-like dissociation between networks for mathematics in parietal cortex and for reading in temporal cortex across multiple measures in early childhood. This is the first demonstration of a functional dissociation between mathematics and reading identified within-subjects in children as young as 4 years of age. That we see this dissociation across both active and passive tasks with naturalistic and controlled stimuli suggests that differences between reading and math are unlikely to be due to domain general factors such as differences in attention and working memory during reading and mathematics processing. Within this adult-like dissociation we found that some regions of the brain showed neural timecourses that were developing or adult-like and others that were “child-unique”. This shows that the subcomponents of these networks develop at different rates, likely reflecting the acquisition of new skills and the abandonment of child-unique strategies that underlie reading and mathematics.

“Child-unique” neural activation has not been studied in depth but is expected based on cognitive development research in which children rely on cognitive strategies that adults do not (e.g., Cornell, 1985; Ehri and McCormick, 1998; Fischer, 2011; Fischer and Koch, 2016; Portex et al., 2018; Siegler, 1996; Siegler and Jenkins, 1989; Vellutino, 1979). To identify “child-unique” neural activity, we applied a novel approach of comparing the synchrony of neural activity across children and across adults using intersubject correlations. We found that in some regions, neural activity associated with spontaneous naturalistic processing of reading and math content was more systematically synchronous across children than across adults, meaning that the patterns of neural activity in those regions are “child-unique”. This type of “child-unique” neural activity is likely to be a distinct form of development that is different from maturation and learning. Whereas maturation and learning are typically associated with a gradual strengthening of adult networks, the patterns of “child-unique” neural activity identified here show a degree of synchrony in childhood that is not seen in adulthood. Instead, this pattern represents a discontinuity across development.

Maturation of some of these “child-unique” regions may be linked to structural maturation of either gray matter or white matter. For instance, we know that cortical thickness is much higher in children in the “child-unique” regions in frontal and temporal cortices (Gogtay et al., 2004; Sowell et al., 2004). We also know that functional maturation of ventrolateral and dorsolateral prefrontal cortex for maintenance of information in working memory coincides with rates of structural maturation (Crone et al., 2006; see also Bunge and Wright, 2007; Bunge and Zelazo, 2006). This shows that there are both functional and structural differences in the “child-unique” regions of the brain. We compared signal-to-noise ratio in children and adults to rule out general effects of structural differences on BOLD estimation, but it remains a possibility that increased similarity among children is driven by structural differences that underscore measures of neural activity. It is likely that some of the similarities and differences in brain function during early childhood vs adulthood are constrained by structural changes across development.

Currently the possibility of capturing the strategic changes that children experience in early childhood might seem out of reach, but by looking at child-to-child and child-to-adult correlations in neural activity during naturalistic reading and mathematics tasks, we can begin to gain traction on studying this important developmental phenomenon. For instance, here we have identified regions of the inferior frontal gyrus that show greater neural similarity between children than between adults. These regions are located within both the reading and math networks and also showed “child-unique” patterns of functional connectivity with regions of prefrontal cortex. Because conceptual change requires cooperation between domain-general executive functions in prefrontal cortex and domain-specific systems of core knowledge, one exciting possibility is that the bilateral inferior frontal gyri play a central role in conceptual development underlying mathematics and reading. Examining changes in cognitive strategies alongside changes in neural activity across development will be important for testing this prediction. Longitudinal paradigms that focus on the acquisition of specific concepts in reading, mathematics, or other areas of cognitive development will be especially informative for understanding the functions of “child-unique” neural synchrony and functional connectivity in conceptual development.

The discovery of “child-unique” neural functions offers a new demonstration of the developmental science axiom “children are not just little adults” (Jean Piaget; Ginsburg and Opper, 1988) and provides an entry point for deciphering the neural basis of conceptual change in early childhood.

## Declaration of Competing Interest

None.
