# Supplementary material for: Developing, mature, and unique functions of the child’s brain in reading and mathematics
Source: Dev Cogn Neurosci. 2019 Jul 26;39:100684. doi: 10.1016/j.dcn.2019.100684 (PMC6886692; doi:10.1016/j.dcn.2019.100684)
Supplement: Supplementary file 1 [file mmc1.pdf]

**Supplements to:**

# Developing, Mature, and Unique Functions of the Child's Brain in Reading and Mathematics

Alyssa J. Kersey, Kathryn-Mary Wakim, Rosa Li, & Jessica F. Cantlon

**Supplement 1. Motion vs Intersubject Correlations.**

As described in the main text, we took several steps to ensure that the identification of "child-unique" and developing patterns of neural activity were not driven by motion artifacts. We used online motion corrected data for our analyses and regressed frame-wise displacement (FD) across children's brains (see Methods 2.4 for more details). To further ensure that the Developing and "Child-Unique" profiles were not artifacts of motion, we conducted a regression analysis in each region. Neural similarity was regressed on translation, rotation, and group (adult-to-adult vs child-to-adult for Developing regions and adult-to-adult vs child-to-child for "Child-Unique" regions). Rotation and translation were calculated on the raw EPI series following Grill-Spector, Golarai, & Gabrieli (2008). If these profiles were driven by group differences in motion, the main effect of group should not be significant when motion is added to the model. Instead, we find that main effects of group persist even when controlling for motion (all p's $< 0.00023$). This indicates that even if there are correlations with motion in these regions, they cannot explain the group-level effects. Boxplots of residualized neural similarity in developing and child-unique regions are shown below in Supplemental Figures 1 and 2.

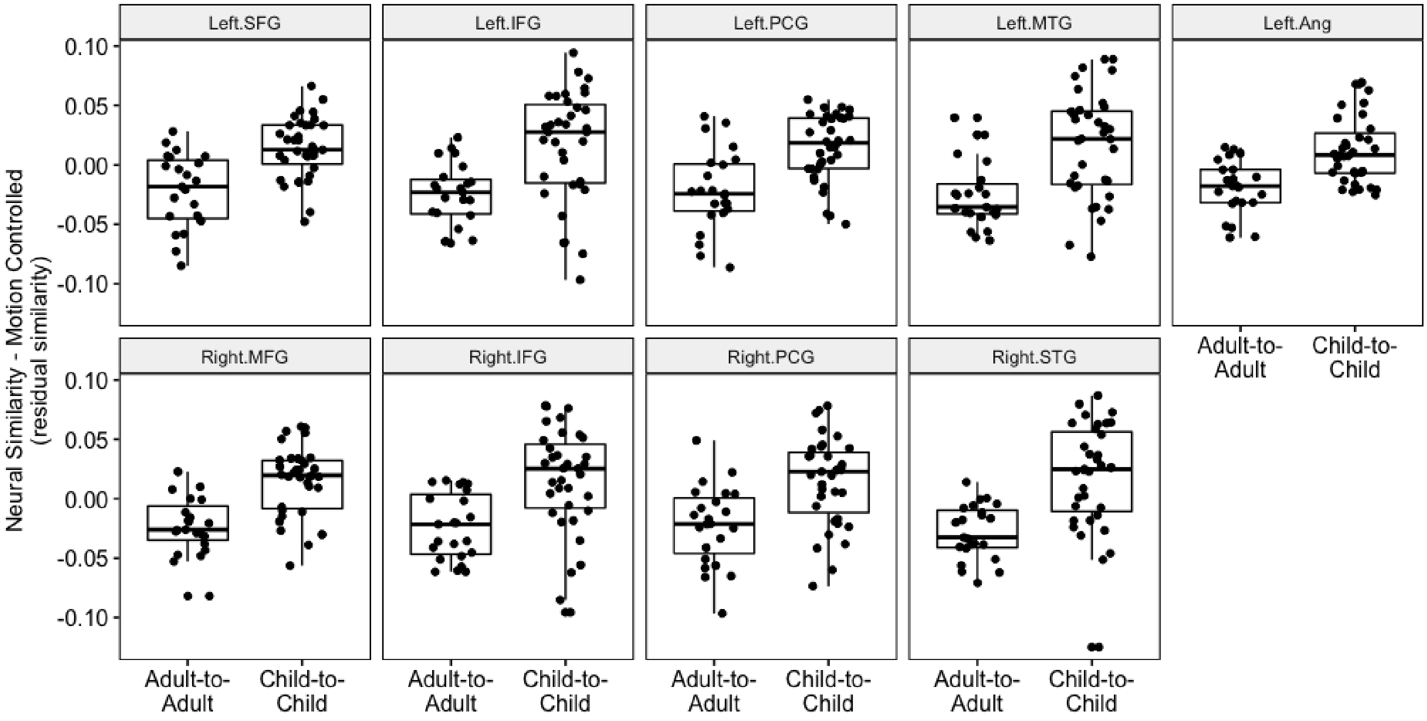


**Supplemental Figure 1. Comparison of within-group similarity controlled for motion in "Child-Unique" regions of the brain.** Regression analyses revealed that group differences between child-to-child similarity and adult-to-adult similarity are evident even when accounting for motion.

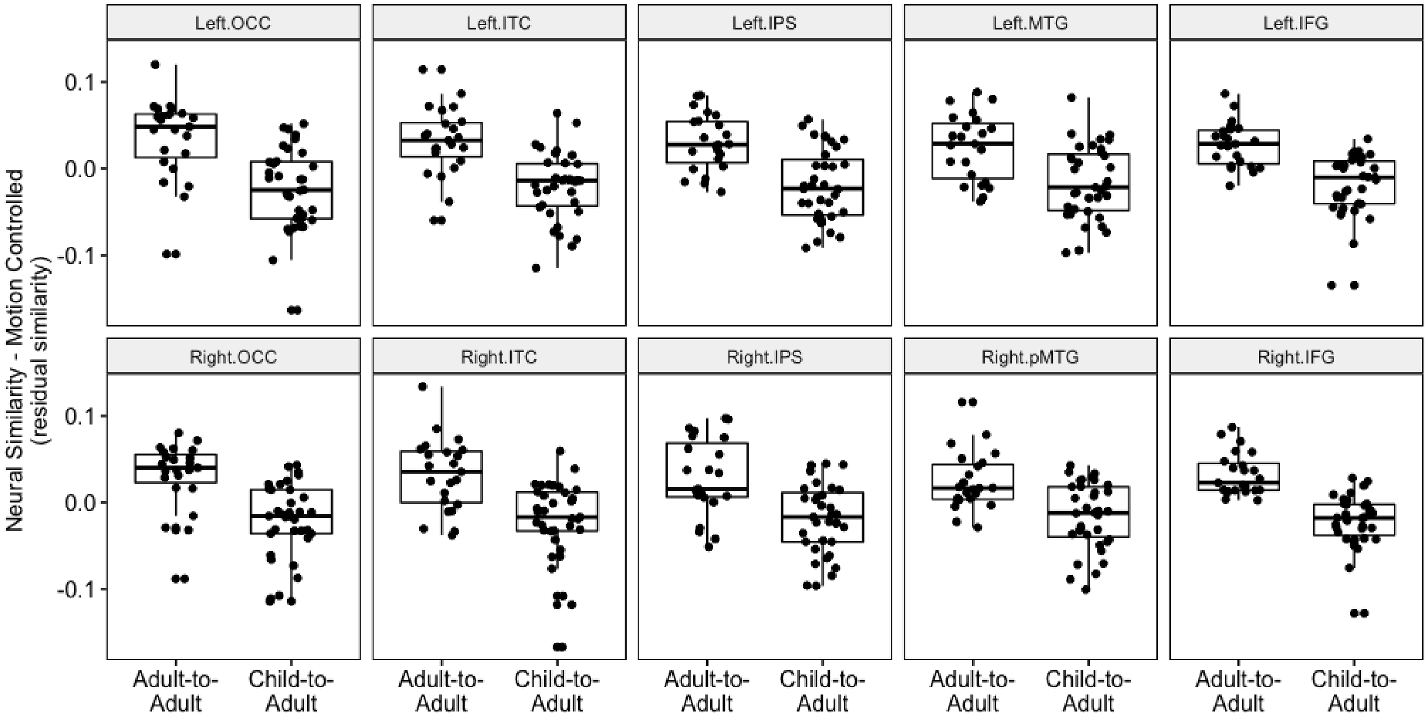


**Supplemental Figure 2. Comparison of adult-to-adult and child-to-adult similarity controlled for motion in "Developing" regions of the brain.** Regression analyses revealed that group differences between child-to-adult similarity and adult-to-adult similarity are evident even when accounting for motion.

## Supplement 2: Table of “Child-Unique” and Developing Regions

List of cortical regions of at least 10 contiguous voxels identified as showing “Child-Unique” or Developing patterns of neural activity during natural viewing of educational video clips. Maximum between group p-value: p = 0.002. *indicates visible on surface rendering in Figure 1

| **Neural Pattern** | **Cortical Region** | **Hemisphere** | **Between Group T-Test** | | | |
|---|---|---|---|---|---|---|
| | | | **Peak X** | **Peak Y** | **Peak Z** | **t(56)** |
| “Child-Unique” | Angular Gyrus* | Left | -54 | -52 | 25 | 6.09 |
| | Postcentral Gyrus* | Left | -42 | -22 | 34 | 6.79 |
| | Postcentral Gyrus* | Right | 48 | -19 | 35 | 5.73 |
| | Inferior Frontal Gyrus* | Left | -33 | 26 | 7 | 5.55 |
| | Inferior Frontal Gyrus | Left | -28 | 11 | -11 | 5.45 |
| | Inferior Frontal Gyrus/Insula* | Right | 27 | 27 | 1 | 7.40 |
| | Inferior Frontal Gyrus | Right | 24 | 11 | -3 | 6.63 |
| | Superior Frontal Gyrus* | Left | -30 | 35 | 30 | 7.06 |
| | Middle Frontal Gyrus* | Right | 24 | 38 | 37 | 7.80 |
| | Paracentral Lobule | Left | -9 | -25 | 49 | 6.22 |
| | Medial Frontal Gyrus | Left | -9 | 15 | 43 | 8.85 |
| | Medial Frontal Gyrus | Bilateral | 0 | 50 | 4 | 6.16 |
| | Amygdala | Left | -21 | -10 | -8 | 6.29 |
| | Middle Temporal Gyrus* | Left | -48 | -16 | -11 | 5.65 |
| | Superior Temporal Gyrus | Left | -51 | -34 | 16 | 4.92 |
| | Superior Temporal Gyrus* | Right | 48 | -10 | -8 | 5.88 |
| | Posterior Superior Temporal Gyrus | Right | 42 | -34 | 10 | 5.31 |
| Developing | Intraparietal Sulcus* | Left | -30 | -61 | 40 | 8.54 |
| | Intraparietal Sulcus* | Right | 18 | -70 | 53 | 7.17 |
| | Inferior Frontal Gyrus* | Left | -42 | 8 | 31 | 6.39 |
| | Inferior Frontal Gyrus* | Right | 48 | -1 | 28 | 7.41 |
| | Superior Temporal Gyrus | Left | -33 | 5 | -26 | 6.52 |
| | Middle Temporal Gyrus* | Left | -43 | -48 | -5 | 5.34 |
| | Middle Temporal Gyrus | Right | 48 | -25 | 11 | 4.35 |
| | Posterior Middle Temporal Cortex* | Right | 57 | -49 | 0 | 5.85 |
| | Inferior Temporal Cortex* | Left | -21 | -43 | -20 | 22.42 |
| | Inferior Temporal Cortex* | Right | 17 | -31 | -21 | 19.11 |
| | Occipital Cortex* | Left | -30 | -82 | 10 | 6.93 |
| | Occipital Cortex | Left | -3 | -61 | -10 | 9.41 |
| | Occipital Cortex | Left | -15 | -64 | 22 | 3.26 |
| | Occipital Cortex* | Right | 24 | -94 | 13 | 7.52 |

## Supplement 3. Table of Regions in Mathematics and Reading Networks

List of cortical regions that comprise the mathematics and reading networks. Regions are listed by analysis and correspond to the results in Section 3.2 and Figure 2. Regions from the ROI analysis are denoted by a * following the region name. Significance of r-values and t-values are denoted following the statistic value by ** p < 0.01,* p < 0.05, † p < 0.10. No symbol indicates p > 0.10.

**Correlations between Math Ability & Neural Maturity (Children Only)**

| Cortical Region | Hemisphere | Peak X | Peak Y | Peak Z | Children: r(30) | | Adults: n/a |
|---|---|---|---|---|---|---|---|
| Intraparietal Sulcus | Left | -12 | -61 | 43 | 0.73 | ** | --- |
| Intraparietal Sulcus | Right | 9 | -64 | 46 | 0.68 | ** | --- |
| Middle Temporal Gyrus | Right | 39 | -37 | 1 | 0.64 | ** | --- |
| Middle Frontal Gyrus | Left | -27 | 35 | -11 | 0.65 | ** | --- |
| Inferior Frontal Gyrus | Right | 24 | 23 | -11 | 0.61 | ** | --- |
| Lingual Gyrus | Left | -24 | -79 | -5 | 0.64 | ** | --- |
| Middle Occipital Gyrus | Right | 24 | -85 | 7 | 0.59 | ** | --- |

**Correlations between Reading Ability & Neural Maturity (Children Only)**

| Cortical Region | Hemisphere | Peak X | Peak Y | Peak Z | Children: r(29) | | Adults: n/a |
|---|---|---|---|---|---|---|---|
| Posterior Parietal Cortex | Left | -9 | -61 | 52 | 0.72 | ** | --- |
| Fusiform Gyrus | Left | -42 | -19 | -20 | 0.60 | ** | --- |
| Middle Temporal Gyrus | Right | 39 | -37 | 1 | 0.72 | ** | --- |
| Inferior Occipital Gyrus | Left | -45 | -76 | -2 | 0.63 | ** | --- |
| Lingual Gyrus | Right | 3 | -70 | -2 | 0.76 | ** | --- |

**Number Localizer (Numbers > Other)**

| Cortical Region | Hemisphere | Peak X | Peak Y | Peak Z | Children: t(25) | | Adults: t(28) | |
|---|---|---|---|---|---|---|---|---|
| Intraparietal Sulcus* | Left | -42 | -49 | 46 | 2.62 | * | 5.42 | ** |
| Intraparietal Sulcus* | Right | 45 | -40 | 43 | 2.25 | * | 5.49 | ** |
| Posterior Parietal Cortex | Left | -21 | -61 | 43 | 3.71 | ** | 4.76 | ** |
| Posterior Parietal Cortex | Right | 18 | -67 | 49 | 3.01 | ** | 6.57 | ** |
| Inferior Frontal Gyrus/Insula* | Left | -33 | 17 | 10 | 1.79 | † | 4.24 | ** |
| Inferior Frontal Gyrus/Insula* | Right | 30 | 20 | 7 | 1.70 | | 3.07 | ** |
| Superior Frontal Gyrus | Right | 24 | 2 | 52 | 0.78 | | 4.32 | ** |
| Anterior Cingulate Cortex | Medial | 3 | 20 | 43 | 1.42 | | 4.34 | ** |

**Word Localizer (Words > Scrambled Words)**

| Cortical Region | Hemisphere | Peak X | Peak Y | Peak Z | Children: t(27) | | Adults: t(28) | |
|---|---|---|---|---|---|---|---|---|
| Middle Temporal Gyrus* | Left | -54 | -37 | 16 | 3.09 | ** | 6.03 | ** |
| Middle Temporal Gyrus* | Right | 45 | -31 | 10 | 2.34 | * | 2.90 | ** |
| Fusiform Gyrus* | Left | -36 | -31 | -17 | 2.85 | ** | 2.66 | * |
| Dorsolateral Prefrontal Cortex | Left | -42 | 23 | 16 | 2.00 | † | 2.20 | * |

| Natural Viewing (Math Clips > Reading Clips) | | | | | | | | |
|---|---|---|---|---|---|---|---|---|
| Cortical Region | Hemisphere | Peak X | Peak Y | Peak Z | Children: t(34) | | Adults: t(22) | |
| Intraparietal Sulcus* | Left | -42 | -64 | 44 | 4.12 | ** | 3.50 | ** |
| Intraparietal Sulcus* | Right | 42 | -58 | 43 | 6.86 | ** | 3.62 | ** |
| Superior Frontal Gyrus | Left | -36 | 54 | 13 | 4.74 | ** | 3.91 | ** |
| Middle Frontal Gyrus | Right | 27 | 11 | 55 | 4.34 | ** | 1.94 | † |
| Lingual Gyrus | Right | 15 | -94 | -5 | 8.04 | ** | 6.05 | ** |

| Natural Viewing (Reading Clips > Math Clips) | | | | | | | | |
|---|---|---|---|---|---|---|---|---|
| Cortical Region | Hemisphere | Peak X | Peak Y | Peak Z | Children: t(34) | | Adults: t(22) | |
| Superior Temporal Gyrus* | Left | -51 | -49 | 10 | 4.05 | ** | 9.46 | ** |
| Superior Temporal Gyrus* | Right | 48 | -40 | 13 | 6.63 | ** | 7.84 | ** |
| Middle Temporal Gyrus* | Left | -51 | -13 | -8 | 4.15 | ** | 4.75 | ** |
| Middle Temporal Gyrus* | Right | 45 | -19 | -5 | 5.87 | ** | 5.88 | ** |
| Fusiform Gyrus* | Left | -39 | -37 | -14 | 1.24 | | 6.14 | ** |
| Anterior Temporal Lobe | Left | -51 | -13 | -8 | 4.15 | ** | 4.75 | ** |
| Anterior Temporal Lobe | Right | 48 | 11 | -17 | 6.94 | ** | 5.80 | ** |
| Inferior Frontal Gyrus* | Left | -45 | 26 | 1 | 5.72 | ** | 7.95 | ** |
| Inferior Frontal Gyrus | Left | -48 | 20 | 19 | 3.89 | ** | 7.33 | ** |
| Inferior Frontal Gyrus* | Right | 48 | 29 | 1 | 5.93 | ** | 4.48 | ** |
| Insula | Left | -27 | -28 | 13 | 4.39 | ** | 4.58 | ** |
| Precentral Gyrus | Left | -36 | -7 | 49 | 2.28 | * | 5.40 | ** |
| Superior Frontal Gyrus | Right | 6 | 47 | 31 | 2.98 | ** | 1.81 | † |
| Superior Frontal Gyrus | Right | 9 | 11 | 58 | 1.72 | † | 1.80 | † |
| Middle Frontal Gyrus | Right | 39 | 11 | 28 | 3.15 | ** | 5.98 | ** |
| Precentral Gyrus | Right | 33 | -7 | 37 | 2.20 | * | 6.30 | ** |
| Precuneus | Left | -6 | -52 | 37 | 4.21 | ** | 2.46 | * |
| Precuneus | Right | 9 | -52 | 34 | 5.01 | ** | 3.24 | ** |

**Supplement 4. Table of Regions that Show "Child-Unique" Functional Connectivity**

List of cortical regions that show strong connectivity among children (r > 0.25) and show greater functional connectivity in children than in adults (t(54) > 2.93, p < 0.005; maximum peak between-group p-value of listed regions: p = 0.00021). Regions from Figure 1 that showed "Child-Unique" patterns of neural activity were used as seed regions. This analysis is described in Section 3.3 and the results are shown in Figure 4. IFG = inferior frontal gyrus, S/MFG = superior/middle frontal gyrus, PCG = precentral gyrus, AG = angular gyrus, STG/MTG = superior/middle temporal gyrus

| Seed Region | Connected Region | | Child > Adult Connectivity | | | | Connectivity (r(346) | |
|---|---|---|---|---|---|---|---|---|
| | Cortical Region | Hemisphere | X | Y | Z | t(54) | Children | Adults |
| Left IFG | Insula | Right | 45 | 8 | 16 | 4.129 | 0.31 | 0.14 |
| | Medial Frontal Gyrus | Right | 15 | 54 | 13 | 6.225 | 0.31 | 0.10 |
| | Medial Frontal Gyrus | Right | 17 | 42 | 21 | 4.723 | 0.38 | 0.20 |
| | Anterior Cingulate | Left | -12 | 29 | 19 | 6.743 | 0.36 | 0.14 |
| | Anterior Cingulate | Left | -15 | 32 | 19 | 6.598 | 0.37 | 0.17 |
| Right IFG | Superior Frontal Gyrus | Right | 21 | 47 | -2 | 4.792 | 0.32 | 0.11 |
| | Inferior Frontal Gyrus | Right | 30 | 29 | -2 | 8.903 | 0.48 | 0.26 |
| | Medial Frontal Gyrus | Left | -18 | 38 | 19 | 6.207 | 0.31 | 0.11 |
| | Anterior Cingulate | Left | -12 | 20 | 25 | 6.678 | 0.35 | 0.14 |
| | Anterior Cingulate | Left | -11 | 29 | 19 | 6.374 | 0.36 | 0.16 |
| | Anterior Cingulate | Right | 18 | 38 | 16 | 5.899 | 0.35 | 0.14 |
| | Anterior Cingulate | Right | 9 | 29 | 4 | 4.8 | 0.32 | 0.13 |
| | Cingulate Gyrus | Right | 9 | 20 | 25 | 5.569 | 0.35 | 0.17 |
| Left SFG | Inferior Frontal Gyrus | Left | -20 | 35 | -5 | 5.6 | 0.28 | 0.05 |
| | Middle Frontal Gyrus | Left | -30 | 29 | 25 | 4.626 | 0.45 | 0.26 |
| | Middle Frontal Gyrus | Right | 27 | 8 | 43 | 4.402 | 0.27 | 0.07 |
| | Medial Frontal Gyrus | Right | 6 | 29 | 46 | 4.921 | 0.31 | 0.12 |
| | Medial Frontal Gyrus | Right | 12 | 53 | 10 | 4.876 | 0.29 | 0.10 |
| | Superior Frontal Gyrus | Left | -15 | 20 | 46 | 4.153 | 0.37 | 0.19 |
| | Superior Frontal Gyrus | Left | -21 | 47 | 16 | 5.207 | 0.39 | 0.17 |
| | Anterior Cingulate | Right | 15 | 37 | 1 | 5.918 | 0.29 | 0.09 |
| | Anterior Cingulate | Right | 15 | 40 | 2 | 5.215 | 0.27 | 0.06 |
| Right MFG | Middle Frontal Gyrus | Left | -33 | 29 | 25 | 4.089 | 0.48 | 0.29 |
| | Middle Frontal Gyrus | Right | 45 | 16 | 25 | 6.667 | 0.30 | 0.05 |
| | Medial Frontal Gyrus | Left | -11 | 53 | 10 | 5.227 | 0.32 | 0.03 |
| | Medial Frontal Gyrus | Right | 21 | 32 | 31 | 4.52 | 0.51 | 0.36 |
| | Medial Frontal Gyrus | Right | 9 | 56 | 7 | 6.982 | 0.33 | 0.08 |
| | Medial Frontal Gyrus | Right | 6 | 57 | 10 | 7.914 | 0.37 | 0.07 |
| | Superior Frontal Gyrus | Right | 15 | 14 | 49 | 4.065 | 0.46 | 0.28 |
| | Superior Frontal Gyrus | Right | 9 | 50 | 38 | 4.005 | 0.35 | 0.08 |
| | Anterior Cingulate | Left | -18 | 29 | 19 | 4.315 | 0.43 | 0.24 |
| | Precentral Gyrus | Right | 31 | -1 | 28 | 4.658 | 0.36 | 0.18 |
| Left PCG | Insula | Left | -36 | -4 | 13 | 4.098 | 0.28 | 0.12 |

| | | | | | | | | |
|---|---|---|---|---|---|---|---|---|
| | Cingulate Gyrus | Left | -24 | -19 | 43 | 4.327 | 0.43 | 0.27 |
| | Precuneus | Left | -24 | -37 | 31 | 6.028 | 0.34 | 0.17 |
| Right PCG | Middle Frontal Gyrus | Right | 51 | 5 | 37 | 4.805 | 0.31 | 0.13 |
| | Medial Frontal Gyrus | Left | -15 | 2 | 49 | 4.195 | 0.29 | 0.12 |
| | Medial Frontal Gyrus | Right | 9 | 2 | 49 | 3.977 | 0.35 | 0.18 |
| | Insula | Left | -33 | -4 | 19 | 5.291 | 0.30 | 0.14 |
| | Insula | Right | 36 | -1 | 22 | 5.88 | 0.34 | 0.18 |
| | Precentral Gyrus | Right | 27 | -22 | 46 | 4.599 | 0.50 | 0.35 |
| Left AG | Superior Temporal Gyrus | Left | -33 | -48 | 16 | 5.929 | 0.35 | 0.16 |
| Left MTG | Superior Temporal Gyrus | Left | -36 | 14 | -29 | 5.352 | 0.28 | 0.12 |
| | Superior Temporal Gyrus | Left | -48 | -31 | 7 | 4.2 | 0.37 | 0.23 |
| Right STG | Superior Temporal Gyrus | Right | 39 | 14 | -26 | 5.038 | 0.31 | 0.16 |

## Supplement 5. Functional Connectivity Analyses with Censored Data

Many studies document that spurious correlations can arise from functional connectivity analyses when the data are contaminated by motion artifacts (e.g., Power et al., 2012; Satterthwaite et al., 2012; van Dijk, Sabuncu, & Buckner, 2012). This is particularly concerning for data obtained from children who move more than adults. Current standards in the field of cognitive neuroscience suggest that the best way to address spurious correlations is to conduct functional connectivity analyses with global signal regression after censoring or “scrubbing” the data (Satterthwaite et al., 2019). The functional connectivity analyses reported in the main text were conducted with global signal regression but frames were not removed. Here, for comparison, we present results of the functional connectivity results for “scrubbed” data. Data were censored by removing the contaminated frame, one frame prior to the contaminated frame, and two frames after the contaminated frame using a contamination threshold of FD > 0.5 (Power et al., 2012). We then compared children and adults by conducting whole-brain t-tests ($t(56) > 2.92$, $p < 0.005$). Supplemental Figure 3 presents the results from the main text and the results from an analysis with the censored data overlaid. Both maps show regions where 1) children show significantly greater functional connectivity to child-unique seed regions than adults and 2) children show strong patterns of functional connectivity ($r(346) > 0.25$). Overall, the results obtained using motion-controlled data as described in the main text and the results obtained using the censored data were nearly identical, suggesting that the controls for motion applied in the main text are as effective as the conventional practice of censoring for this dataset.

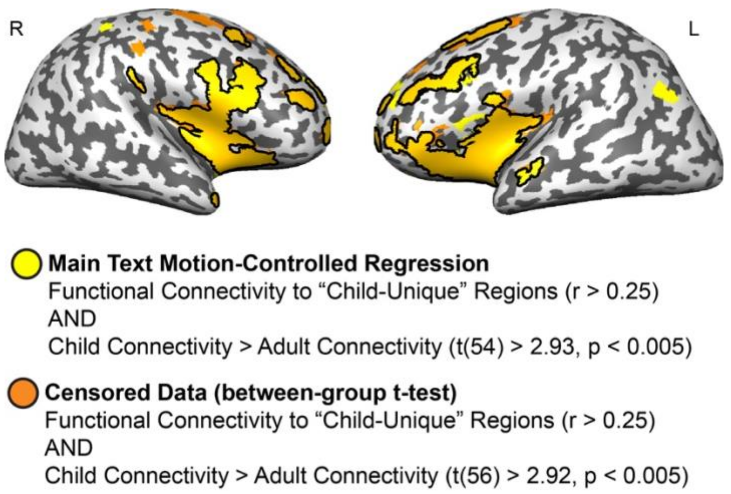


**Supplemental Figure 3. Comparison of Child-Unique Functional Connectivity** between regression analysis controlling motion (yellow, main text) and between-group t-test performed on censored data (orange). The regions of overlap are outlined. Results are displayed with a cluster threshold of 50 $mm^2$
